# Cingulate sulcus sign: a descriptive analysis in a cerebral small vessel disease population

**DOI:** 10.3389/fnagi.2024.1438796

**Published:** 2024-08-06

**Authors:** Weishuai Li, Chang Su, Zhihan Wang, Xiaoxuan Xu, Dongming Zheng

**Affiliations:** Department of Neurology, Shengjing Hospital of China Medical University, Shenyang, Liaoning, China

**Keywords:** cingulate sulcus sign, cerebral small vessel disease, glymphatic system, white matter lesions, cerebral microbleeds

## Abstract

**Objective:**

The cingulate sulcus sign (CSS) has been observed in patients with idiopathic normal pressure hydrocephalus (iNPH), suggesting potential disruptions in cerebrospinal fluid circulation and compromised glymphatic system. Although there are similarities in the underlying mechanisms between cerebral small vessel disease (CSVD) and iNPH, the relationship between CSS and CSVD remains unclear. This study aimed to investigate the prevalence and potential mechanisms of CSS in patients with CSVD.

**Methods:**

Data from patients diagnosed with CSVD at Shengjing Hospital of China Medical University between January 2020 and October 2022 were retrospectively collected, including general information, global cognitive function [assessed by measuring Mini-Mental State Examination (MMSE)], and four CSVD magnetic resonance imaging (MRI) markers [(white matter hyperintensity (WMH), cerebral microbleeds (CMBs), lacunes, and enlarged perivascular spaces (EPVS)], CSS and the Evan’s index (EI).

**Results:**

A total of 308 patients were included, and CSS was detected in 80 patients (26%). Univariate analysis revealed that MMSE scores in the CSS group were significantly lower compared to the non-CSS group (*p* < 0.001). Multivariable analysis showed an independent correlation between CSS and the presence of lacunes (odds ratio [OR] 0.358, 95% confidence interval [CI] 0.193–0.663, *p* = 0.001), presence of lobar dominant CMBs (OR 2.683, 95%CI 1.385–5.195, *p* = 0.003), periventricular WMH Fazekas score (OR 1.693, 95% CI 1.133–2.529, *p* = 0.01), and EI (OR 1.276, 95% CI 1.146–1.420, *p* < 0.001).

**Conclusion:**

This preliminary study showed that CSS can be observed in some patients with CSVD. The presence of CSS may represent different mechanisms of CSVD pathogenesis and reflect differences in the degree of cerebrospinal fluid (CSF)/interstitial fluid (ISF) stasis.

## 1 Introduction

The cingulate sulcus is a prominent cerebral sulcus situated on the medial surface of the cerebral hemispheres. It originates in the medial portion of the frontal lobe and progresses posteriorly upward to the crest of the hemisphere’s medial surface, creating a boundary between the paracentral lobule and the precuneus (Allison et al., 1996). Notably, the posterior segment of the cingulate sulcus exhibits greater variability than its anterior counterpart, owing to its later development during embryogenesis. Consequently, age-related dilation of the cingulate sulcus is predominantly observed within its posterior portion (Naidich et al., 1994). Researchers have surprisingly discerned a particular morphological variant termed the cingulate sulcus sign (CSS), characterized by an expanded anterior cingulate relative to the posterior cingulate, among some patients with idiopathic normal pressure hydrocephalus (iNPH) who are deemed suitable for ventriculoperitoneal shunt surgery (Otero-Rodríguez et al., 2019). Current understanding implicates a compromised glymphatic system in the pathophysiology of iNPH (Andersson et al., 2017; Ringstad et al., 2017). This system collaborates with both arterial pulsatility and venous compliance to facilitate the flow of cerebrospinal fluid (CSF) through brain tissue and the extraction of solutes from the interstitial fluid (ISF), which is essential for the upkeep of tissue balance and metabolic stability (Iliff et al., 2012). Since the gold standard diagnosis of this disease is a favorable response to ventriculoperitoneal shunt surgery (Andersson et al., 2017), we speculate that the presence of CSS in iNPH patients may indicate altered CSF dynamics and suggest dysfunction of the glymphatic system. As the flow of CSF heavily rely on cardiocirculatory and vascular factors, any impairment, including those of arterial pulsatility, microvascular changes, or decreased venous drainage, may contribute to dysfunction of the glymphatic system in the brain (Iliff et al., 2013; Mestre et al., 2018; van Veluw et al., 2020). Cerebral small vessel disease (CSVD) is a group of cerebrovascular diseases that primarily affect perforating arteries, capillaries or leptomeningeal vessels with various neuropathies (Pantoni, 2010; Charidimou et al., 2015), and cause a wide range of clinical manifestations, including stroke, cognitive impairment, and motor and gait impairment (Pasi and Cordonnier, 2020). The clinical diagnosis of CSVD is primarily through magnetic resonance imaging (MRI) of parenchymal lesions corresponding to small cerebral arteries, which include recent small subcortical infarcts, lacunes of presumed vascular origin, white matter hyperintensities (WMH) of presumed vascular origin, enlarged perivascular spaces (EPVS), cerebral microbleeds (CMBs) and brain atrophy (Wardlaw et al., 2013). Indeed, an increasing number of studies have shown that patients with CSVD suffer from damage of the glymphatic system (Xue et al., 2020; Zhang et al., 2021; Tang et al., 2022; Xu et al., 2022). Based on the hypothesis that CSS may represent impaired CSF circulation and glymphatic system dysfunction, we anticipate the presence of CSS in CSVD as well. Therefore, this study aimed to investigate the prevalence of CSS in patients with CSVD and attempt to elucidate potential glymphatic system disorders associated with CSS by comparing the degree of CSF/ISF sludge. To the best of our knowledge, there have been no previous studies on this subject.

## 2 Materials and methods

### 2.1 Study population and data collection

Patients who visited the clinic at the Neurology Department of Shengjing Hospital and met the diagnosis of CSVD were retrospectively enrolled in our study consecutively from January 2020 to October 2022. Subjects were enrolled if they met the following criteria: (1) Their clinical symptoms, signs and MRI findings met the diagnostic criteria for CSVD (Wardlaw et al., 2013), (2) CT or MRI excluding ICH; (3) they had a complete set of MRI images including T1-weighted, T2-weighted, and diffusion-weighted imaging (DWI), susceptibility weighted imaging (SWI), fluid-attenuated inversion recovery (FLAIR) imaging and magnetic resonance arteriography (MRA). The following were the exclusion criteria: (1) stroke from any potential cardioembolic source; (2) genetically confirmed hereditary CSVD or suspected hereditary CSVD based on family history and clinical features; (3) WMH that could be caused by something other than CSVD, such as multiple sclerosis; (4) patients suspected comorbidity include iNPH (Evan’s index (EI) ≥ 0.3 or disproportionately enlarged subarachnoid-space hydrocephalus), Alzheimer’s disease, or Parkinson’s disease.

Demographic and clinical information was obtained by a review of the medical records or interviews with the patients or their family members. The presence of hypertension, hyperlipidemia, diabetes and previous stroke was determined based on a prior medical diagnosis and treatment among all patients. Smoking or drinking was defined by a history of tobacco or alcohol use. Meanwhile, all patients underwent the Mini-Mental State Examination (MMSE) as a measure of global cognitive function. The present study was performed in accordance with the ethical standards established in the 1964 Declaration of Helsinki and its subsequent amendments. The ethics committee of our institution approved the procedures of this study.

### 2.2 Neuroimaging acquisition and analysis

**FIGURE 1:**
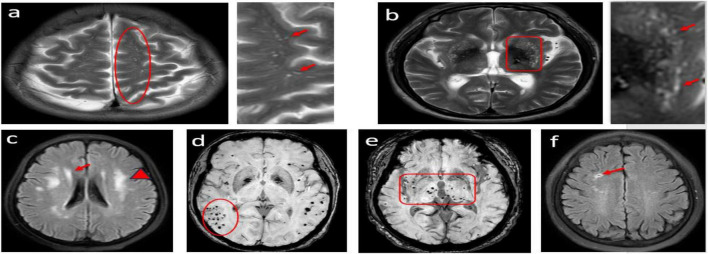
Cases of MRI findings for lesions related to small vessel disease; **(a)** Axial T2-weighted MRI showing punctuate and linear hyperintensities characteristic of enlarged perivascular spaces (EPVS) in the centrum semiovale. **(b)** punctuate hyperintensities characteristic of EPVS in the basal ganglia. **(c)** Deep white matter hyperintensity (WMH) graded as 3 (red arrowheads) in a patient who also has periventricular WMH (graded as 2, red arrows). **(d,e)** different topographic distributions cerebral microbleeds (lobar/deep). **(f)** lacunes of presumed vascular origin.

Conventional imaging was obtained with a 3.0T MR scanner including T2-weighted, T1-weighted and FLAIR. SWI acquisition was performed with a T2*-weighted gradient echo sequence with a flip angle of 15°, TR/TE = 30/20 ms, and slice thickness = 1.8 mm. SWI and minimum intensity projection images were acquired by in-line post processing of magnitude and phase images, and these post processed images were used for the evaluation of the imaging findings. EPVS, CMB, lacune and WMH were defined according to the Standards for Reporting Vascular changes on Euroimaging (Wardlaw et al., 2013). Representative images of characteristic CSVD lesions are shown in Figures 1a–f. The number of EPVS was measured in the basal ganglia (BG) and centrum semiovale (CSO) and was dichotomized as either high degree (score > 20) or low degree (score ≤ 20) according to a previously published protocol (Charidimou et al., 2017a). The number of CMBs was measured in lobar and/or deep and was categorized into the lobar dominant group and non-lobar dominant group according to the ratio of lobar to deep CMBs number. The WMH were scored on FLAIR images using the Fazekas score (Kim et al., 2008).

The EI was defined as the ratio between the maximal width of the frontal horns of the lateral ventricles and the maximal inner diameter of the skull on the same slice (Relkin et al., 2005).

**FIGURE 2:**
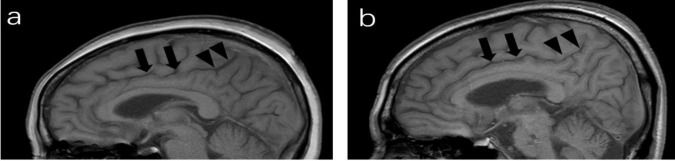
Paramedian sagittal T1-weighted image of MRI; **(a)** a 64-year-old man (negative), the posterior part (arrowheads) of the cingulate sulcus is slightly wider than the anterior part (arrows). **(b)** cingulate sulcus sign (CSS): a 67-year-old man, the anterior part of the cingulate sulcus are wide (arrows), whereas the posterior part is tight (arrowheads).

CSS were identified on paramedian-sagittal T1 weighted images according to a previously published protocol (Otero-Rodríguez et al., 2019). It was divided into anterior and posterior parts by a line that included pontomedullary junction parallel to the fourth ventricle floor. A normal finding was defined as the anterior part of the cingulate sulcus being the same width as the posterior part or the posterior part being wider than the anterior part (Figure 2a). Positive CSS being noticeably narrower in the posterior part compared to the anterior part (Figure 2b). The kappa coefficient for CSS was 0.88 (*p* < 0.001).

The above image features were identified and counted by two experienced researchers independently, and the researchers were blinded to the patients’ clinical data. In the event of any disagreement between the two raters, a consensus was reached with the help of a neuroradiologist (D.M.Z).

### 2.3 Statistics

Data normality was assessed using the Kolmogorov–Smirnov test. Continuous variables are represented as interquartile ranges (IQRs). Categorical variables were described using frequencies (N, %). Univariate analyses were performed to evaluate differences in demographic, clinical, and neuroimaging characteristics between the patients with and without CSS using χ^2^ and Fisher’s exact tests for categorical variables and Mann–Whitney U tests for continuous variables. To test for independent associations with the presence of CSS, all imaging variables that showed a *p*-value < 0.05 in the univariate analyses were entered in a multivariable logistic regression analysis. Logistic regression models were run with a stepwise forward elimination method to generate a minimal adjusted model. In this study, for purposes of statistical analyses, we convert continuous data EI into ordinal data using the fixed interval method, categorizing the data based on a fixed interval of 0.01. All statistical analyses in this study were performed with SPSS 22.0 software (IBM SPSS Inc., Chicago, IL, United States), and statistical significance was set at *p* < 0.05.

## 3 Results

**FIGURE 3:**
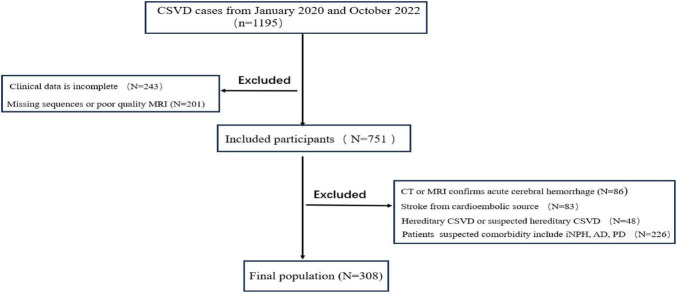
Flow chart of this study.

Figure 3 shows the flow chart of this study. A total of 308 patients with CSVD were included in the study. The average age of the patients was 66.0 (61.0–72.0) years. Approximately 55.2% of the patients were male. The presence of lacunes and CMBs, lobar dominant CMBs, high degree BG-EPVS and high degree CSO-EPVS, were observed in 187 (60.7%), 131 (42.5%), 82 (26.6%), 135 (43.8%), and 166 (53.9%) patients, respectively. The median scores for deep WMH, periventricular WMH, EI and MMSE were 2.0 (1.0–3.0), 3.0 (2.0–3.0), 0.25 (0.23–0.27) and 27 (26–29) respectively.

### 3.1 Univariable analysis of clinical and imaging characteristics of study population

The basic characteristics of the CSS group and non-CSS group are listed in Table 1. Except for MMSE scores (*p* < 0.001), there were no significant differences in demographic and clinical information between the two groups (all *p* > 0.05). The presence of lacunes was significantly lower in the CSS group compared to the non-CSS group (*p* = 0.023). Moreover, the CSS group displayed a higher EI (*p* < 0.001), more severe deep WMH (*p* = 0.001) and periventricular WMH (*p* < 0.001), a higher prevalence of lobar dominant CMBs (*p* = 0.02) and high degree CSO-EPVS (*p* = 0.01). However, no significant difference was observed in high degree BG-EPVS and presence of CMBs between the two groups.

**TABLE 1:** Comparison of demographic, clinical, and neuroimaging characteristics between patients with non-CSS and CSS.

Variable	Total (*n* = 308)	non-CSS (*n* = 228)	CSS groups (*n* = 80)	*P*-value (non-CSS vs. CSS)
Age median (IQR)	66.0 (61.0–72.0)	66.0 (61.0–72.0)	66.0 (61.0–73.0)	0.907
Sex, (male) (*N*%)	170 (55.2%)	120 (52.6%)	50 (62.5%)	0.127
Hypertension (*N*%)	198 (64.3%)	141 (61.9%)	57 (71.3%)	0.131
Hypercholesterolemia (*N*%)	84 (27.3%)	57 (25.0%)	27 (33.8%)	0.131
Type 2 diabetes (*N*%)	78 (25.3%)	53 (23.2%)	25 (31.3%)	0.157
Smoking habits (*N*%)	110 (35.7%)	79 (34.6%)	31 (38.6%)	0.51
Drinking habits (*N*%)	97 (31.5%)	70 (30.7%)	27 (33.8%)	0.614
History hemorrhagic stroke (*N*%)	53 (17.2%)	34 (14.9%)	19 (23.6%)	0.072
History of TIA or stroke (*N*%)	105 (34.1%)	72 (31.6%)	33 (41.3%)	0.116
Aspirin use (*N*%)	99 (32.1%)	70 (30.7%)	29 (36.3%)	0.361
MMSE score median (IQR)	27 (26–29)	29 (28–30)	27 (26–28)	<0.001
Lacunes (*N*%)	187 (60.7%)	147 (64.5%)	40 (50.0%)	0.023
CMBs (*N*%)	131 (42.5%)	96 (42.1%)	35 (43.8%)	0.798
Lobar dominant CMBs (*N*%)	82 (26.6%)	50 (21.9%)	32 (40.0%)	0.02
High degree BG-EPVS (*N*%)	135 (43.8%)	98 (43.0%)	37 (46.3%)	0.612
High degree CSO-EPVS (*N*%)	166 (53.9%)	113 (49.6%)	53 (66.3%)	0.01
Deep WMH Fazekas score median (IQR)	2.0 (1.0–3.0)	2.0 (1.0–3.0)	2.0 (1.0–3.0)	0.001
Periventricular WMH Fazekas score median (IQR)	3.0 (2.0–3.0)	2.0 (2.0–3.0)	3.0 (2.0–3.0)	<0.001
Evan’s index median (IQR)	0.25 (0.23–0.27)	0.24 (0.22, 0.27)	0.27 (0.25–0.28)	<0.001

CMBs, cerebral microbleeds; CSO-EPVS, centrum semiovale enlarged perivascular spaces; BG-EPVS, basal ganglia enlarged perivascular spaces; WMH, white matter hyperintensity; CSS, cingulate sulcus sign.

### 3.2 Multivariable analysis of neuroimaging markers associated with CSS

The multivariable analysis of the association between imaging markers of CSVD and CSS is presented in Table 2. After adjustment for variables that were significant in the univariate analysis, CSS was independently correlated with presence of lacunes (odds ratio [OR] 0.358, 95% confidence interval [CI] 0.193–0.663, *p* = 0.001), presence of lobar dominant CMBs (OR 2.683, 95% CI 1.385–5.195, *p* = 0.003), periventricular WMH Fazekas score (OR 1.693, 95% CI 1.133–2.529, *p* = 0.01) and EI (OR 1.276, 95% CI 1.146–1.420, *p* < 0.001).

**TABLE 2:** Multivariable logistic regression analyses of associations with CSS.

Predictor	CSS	
	OR (95% CI)	*P*-value
Lacunes	0.358 (0.193–0.663)	0.001
Lobar dominant CMBs,	2.683 (1.385–5.195)	0.003
Periventricular WMH Fazekas score	1.693 (1.133–2.529)	0.01
Evan’s index	1.276 (1.146–1.420)	<0.001

CMBs, cerebral microbleeds; WMH, white matter hyperintensities; CSS, cingulate sulcus sign.

## 4 Discussion

In this paper, we demonstrate that approximately 26% (80/308) of patients with cerebral small vessel disease (CSVD) exhibit the cingulate sulcus sign (CSS). There were no significant differences in demographics and vascular risk factors between the CSS and non-CSS groups. However, imaging findings revealed significant differences between the two groups in the presence of lacunes, lobar dominant cerebral microbleeds (CMBs), high degrees of centrum semiovale enlarged perivascular spaces (CSO-EPVS), Evan’s index (EI), and Fazekas scores for white matter hyperintensities (WMH). Additionally, the CSS group had significantly lower MMSE scores compared to the non-CSS group. Dominant lobar cerebral CMBs, periventricular WMH, and EI were predictors of CSS, whereas lacunes were more frequently associated with the typical CSVD pattern of cingulate gyrus atrophy.

The cingulate sulcus as an important brain anatomical structure, is widely used in the study of various neurological disorders, and the degree of its atrophy is related to the progression of the disease (Jouvent et al., 2008; Reiner et al., 2012). However, most of these studies employed computerized automated voxel morphometric analyses, which do not cater to individual assessment. Recently, a semi-quantitative visual method was used to demonstrate that CSS may potentially represent disorders of cerebrospinal fluid (CSF) circulation and the glymphatic system in patients with idiopathic normal pressure hydrocephalus (iNPH) (Otero-Rodríguez et al., 2019). In our current exploration, we observed the presence of CSS in some CSVD patients. The observations are consistent with earlier study findings which suggest that atherosclerosis and small vessel stiffness lead to reduced vascular pulsation and subsequently result in weakening CSF/interstitial fluid (ISF) exchange (Iliff et al., 2013; Mestre et al., 2018; van Veluw et al., 2020). Further research has also shown a link between impaired glymphatic function and the development of cognitive impairment in CSVD patients (Tang et al., 2022; Xu et al., 2022). This finding aligns with our discovery that the CSS group exhibits more severe global cognitive impairment, as indicated by lower MMSE scores. Even though a healthy control group was not considered in this study, the similarity in age and vascular risk factors between the two groups suggest that the emergence of CSS is not merely a result of aging or atherosclerosis, but may be linked to different pathogenic mechanisms of CSVD, such as cerebral amyloid angiopathy (CAA), which is typically characterized by the accumulation of amyloid β in the walls of leptomeningeal vessels and cortical capillaries in the brain (Charidimou et al., 2017b).

EI is frequently used as a quick and easy way to evaluate ventriculomegaly (Relkin et al., 2005), which can either be secondary to brain atrophy or represent an impairment of CSF circulation. In this study, we revealed a strong association between the presence of CSS and a larger EI. To minimize the interference of iNPH, we specifically selected patients with an EI of less than 0.3 for this study, which might have reduced the power to detect associations. Nevertheless, we were still able to find significant associations between CSS and EI. However, a automated surface-based study on the morphology of the cingulate sulcus found that patients with vascular cognitive disorders, exhibiting widened cingulate sulcus and accompanying ventriculomegaly (Kuchcinski et al., 2019). One possible explanation for the contradictory results is that CSS is different from the mechanism underlying computer-automated morphological changes in the cingulate sulcus. Unlike the latter, CSS represents impaired CSF circulation rather than being solely secondary to cerebral atrophy. Furthermore, our research has uncovered a greater prevalence of lacunar within the non-CSS group. This can be attributed to the relationship between lacunar infarcts and brain atrophy. Previous studies have demonstrated that lacunar infarcts are associated with a greater progression of brain atrophy and cortical thickness in patient of CSVD (Kloppenborg et al., 2012). Notably, these pathological atrophies are particularly pronounced in the posterior region (Naidich et al., 1994; Reiner et al., 2012). This form of atrophy is consistent with the morphological characteristics of the non-CSS group in this study.

In the present study, we found that CSS was mostly present in CSVD patients with predominantly lobar CMBs. CMBs are small, round collections of hemosiderin-laden macrophages in the brain that represent areas of previous microhemorrhages (Haller et al., 2018), They are now considered an imaging marker for CSVD (Pantoni, 2010). Previous studies have reported that different topographic distributions of CMBs indicate different pathological mechanisms. For instance, CMBs in deep brain regions are radiological biomarkers of hypertensive arterial pathology (Romero et al., 2014), whereas CMBs in superficial or predominantly lobar regions are strongly associated with CAA pathology (Tsai et al., 2017; Greenberg and Charidimou, 2018). Unlike hypertension-related CSVD, CAA is an important risk factor for spontaneous intracerebral hemorrhage and antiplatelet drugs should be discontinued to reduce the risk of hemorrhage (Vales-Montero et al., 2019). Our results suggest that the emergence of CSS may be related to CAA, and therefore, CSS seems to be useful in formulating secondary prevention strategies for CSVD patients. Additionally, the imaging characteristics of CSS mentioned in this study are similar to those observed by Wang et al. (2020) and Beaman et al. (2022) who found that the presence of superficial CMBs can lead to an increase in white matter volume in the parietal and occipital regions due to impaired glymphatic system function, resulting in a narrower posterior part of the cingulate sulcus compared to the anterior part.

WMH of presumed vascular origin are frequent in cerebral MRI of older people (Kim et al., 2008; Valdes Hernandez Mdel et al., 2013). Our data suggest that despite increased WMH scores in the CSS group, there was no difference in vascular risk factors, including hypertension, which suggests that the mechanism underlying WMH may be multifactorial. This conclusion is supported by previous research, indicating that WMHs are promoted by vascular risk factors, especially hypertension and usually reflect axonal loss and demyelination following chronic ischemia (van Veluw et al., 2020). They also result from the failure to eliminate ISF from the white matter (Prins and Scheltens, 2015; Tang et al., 2022). In contrary to deep WMH, which may be impacted by both ischemia-hypoperfusion and malfunction of the glymphatic pathway, periventricular WMH is primarily linked to glymphatic pathway dysfunction (Cai et al., 2022). Based on the aforementioned studies, the independent association between periventricular WMH and CSS in our study strengthens the notion that disturbances of the glymphatic system may be linked to CSS.

Previous studies have shown that despite overlapping risk factors, the different spatial distributions of EPVS represent distinct pathological mechanisms of CSVD. CSO-EPVS suggests CAA whereas basal ganglia enlarged perivascular spaces (BG-EPVS) is associated with hypertensive vasculopathy (Charidimou et al., 2017a; Wardlaw et al., 2020). In the present study, the CSS group had a higher occurrence of CSO-EPVS, which is in line with our hypothesis that CSS may represent an imaging change associated with CAA. Recent studies on CSF circulation disorders have linked EPVS in CSVD to the glymphatic system (Moretti and Caruso, 2020). However, there are conflicting reports regarding the association of the topographic distribution of EPVS and the glymphatic system, One study on the pathology of CAA showed that CSO-EPVS were associated with glymphatic failure (van Veluw et al., 2016), and two clinical studies found glymphatic failure were associated with BG-EPVS evaluated by diffusion tensor image analysis along the perivascular space (Zhang et al., 2021; Xu et al., 2022). This discrepancy may be related to different study populations and methods of assessing the glymphatic system. In our study, we were unable to replicate the above findings. This raises the possibility that CSS, as a semi-quantitative visual imaging marker, may not be sensitive enough to indicate impaired glymphatic system function. The blood-brain barrier (BBB), similar to the glymphatic system, involves pathophysiological aspects such as vascular risk factors, white matter hyperintensities, CSF dynamic disorders, and EPVS. In recent years, various magnetic resonance techniques have been developed to measure the BBB leakage and the BBB water exchange rate (Uchida et al., 2023). Studies have identified heterogeneity in BBB damage patterns in different types of CSVD using magnetic resonance techniques. Hereditary CSVD is characterized by a decrease in water exchange rate, whereas sporadic CSVD shows increased leakage (Ying et al., 2024). Our study found that the presence of CSS may indicate different mechanisms of CSVD pathogenesis, suggesting potentially distinct BBB damage patterns. Further investigation is needed to confirm this hypothesis.

## 5 Limitations

There are also a few limitations to consider. First, our study had a retrospective design and a relatively small sample size, the participants in our study were symptomatic CSVD patients, which limits the ability to establish a causal relationship between CSS and imaging markers. To validate this relationship, future research should include a larger cohort of CSVD patients and normal controls, employ a longitudinal study design; Second, the relationship between CSS and glymphatic system and CSF circulation disorders is mainly based on indirect inferences, lacking direct imaging evidence; and finally, due to the lack of pathological evidence, we were unable to solely rely on clinical and imaging manifestations to differentiate CSVD from conditions like iNPH, AD or PD. This may have exaggerated the relationship between CSS and CSVD.

## 6 Conclusion

In this study, we examined the relationship between CSS and CSVD for the first time. The findings presented here suggest that the presence of CSS in CSVD patients may represent a different etiology and indicate some degree of glymphatic system disorder. Future longitudinal studies using advanced magnetic resonance imaging technologies will provide further insights into the relationship between CSS and the glymphatic system in CSVD, enhancing our understanding of the etiology and clinical relevance of this marker.

## Data availability statement

The original contributions presented in the study are included in the article/supplementary material, further inquiries can be directed to the corresponding author.

## Ethics statement

The studies involving humans were approved by the Ethics Committee of Affiliated Shengjing Hospital of China Medical University. The studies were conducted in accordance with the local legislation and institutional requirements. The ethics committee/institutional review board waived the requirement of written informed consent for participation from the participants or the participants’ legal guardians/next of kin because Due to the retrospective nature of the study and the fact that not all patients could be contacted, and the study process did not involve any projects that could disclose patients’ personal privacy.

## Author contributions

WL: Writing–review and editing, Project administration, Writing–original draft, Supervision, Investigation, Formal analysis, Conceptualization. CS: Writing–original draft, Software, Data curation. ZW: Writing–original draft, Methodology, Data curation. XX: Writing–original draft, Software, Investigation. DZ: Methodology, Writing–review and editing, Visualization, Supervision, Resources, Project administration, Funding acquisition, Conceptualization.
